# MSCAN: multi-scale self- and cross-attention network for RNA methylation site prediction

**DOI:** 10.1186/s12859-024-05649-1

**Published:** 2024-01-17

**Authors:** Honglei Wang, Tao Huang, Dong Wang, Wenliang Zeng, Yanjing Sun, Lin Zhang

**Affiliations:** 1 School of Information and Control Engineering, China University of Mining and Technology, Xuzhou, 221116 China; 2 School of Computer Science and Technology, China University of Mining and Technology, Xuzhou, 221116 China; 3 School of Information Engineering, Xuzhou College of Industrial Technology, Xuzhou, 221400 China

**Keywords:** RNA methylation, Transformer, Predictor, Multi-scale, Self-attention, Cross-attention

## Abstract

**Background:**

Epi-transcriptome regulation through post-transcriptional RNA modifications is essential for all RNA types. Precise recognition of RNA modifications is critical for understanding their functions and regulatory mechanisms. However, wet experimental methods are often costly and time-consuming, limiting their wide range of applications. Therefore, recent research has focused on developing computational methods, particularly deep learning (DL). Bidirectional long short-term memory (BiLSTM), convolutional neural network (CNN), and the transformer have demonstrated achievements in modification site prediction. However, BiLSTM cannot achieve parallel computation, leading to a long training time, CNN cannot learn the dependencies of the long distance of the sequence, and the Transformer lacks information interaction with sequences at different scales. This insight underscores the necessity for continued research and development in natural language processing (NLP) and DL to devise an enhanced prediction framework that can effectively address the challenges presented.

**Results:**

This study presents a multi-scale self- and cross-attention network (MSCAN) to identify the RNA methylation site using an NLP and DL way. Experiment results on twelve RNA modification sites (m^6^A, m^1^A, m^5^C, m^5^U, m^6^Am, m^7^G, Ψ, I, Am, Cm, Gm, and Um) reveal that the area under the receiver operating characteristic of MSCAN obtains respectively 98.34%, 85.41%, 97.29%, 96.74%, 99.04%, 79.94%, 76.22%, 65.69%, 92.92%, 92.03%, 95.77%, 89.66%, which is better than the state-of-the-art prediction model. This indicates that the model has strong generalization capabilities. Furthermore, MSCAN reveals a strong association among different types of RNA modifications from an experimental perspective. A user-friendly web server for predicting twelve widely occurring human RNA modification sites (m^6^A, m^1^A, m^5^C, m^5^U, m^6^Am, m^7^G, Ψ, I, Am, Cm, Gm, and Um) is available at http://47.242.23.141/MSCAN/index.php.

**Conclusions:**

A predictor framework has been developed through binary classification to predict RNA methylation sites.

## Background

RNA modification plays a fundamental role in regulating RNA function [1] and has become a hotspot in epigenetics research [2]. Nearly 200 RNA modifications have been discovered, most of which are methylation modifications [3]. Common RNA methylation types include N^1^-methyladenosine (m^1^A), N^2^-methylguanosine (m^2^G), 5-methylcytosine (m^5^C), 5-methyluridine (m^5^U), 2′-*O*-methyladensine (Am), 2′-*O*-methylcytidine (Cm), 2′-*O*-methylguanosine (Gm), 2′-*O*-methyluridine (Um), Pseudouridine (Ψ), N^6^-methyladenosine(m^6^A), N^7^-methylguanine (m^7^G), inosine (I), and N6,2′-*O*-dimethyladenosine(m^6^Am), etc. Among them, m^6^A refers to methylation modification occurring at the nitrogen atom in position 6 of the RNA molecule adenine, which is the most abundant mRNA methylation, and is known to affect mRNA stability, splicing, and translation. In addition to m^6^A, m^1^A RNA methylation is a recently discovered one, which is evolutionarily conserved and ubiquitous in humans, rodents, and yeast. It can significantly enhance the protein translation of transcripts [4], block the Watson–Crick interface, and is essential for tRNA stability [5].

In the last decade, dozens of experimental methods have been developed to identify the precise location of methylation sites on RNA, such as miCLIP [6], m^1^A-seq [7], PA-m6A-seq [8], m^1^A-ID-seq [9], m5C-RIP [10], m^1^A-MAP [11], and m^1^A-IP-seq [12]. Despite their effectiveness, these experimental techniques are usually both time-consuming and costly, limiting their use in different biological contexts [4], and making them inadequate for large-scale genomic data [13]. Consequently, there is strong motivation to explore computational methods that can accurately and efficiently identify methylation sites based on sequence information alone.

As there are more vailable base-resolution datasets, researchers have designed some computational methods for RNA modification site prediction. These approaches formulate RNA methylation identification as a binary prediction task, and some machine learning models are trained to distinguish between truly methylated and non-methylated sites. These computational methods have been powerful additions for RNA methylation site prediction.

Traditional methods designed for sequence-based prediction usually first extract features based on human-understandable feature methods and then use a classifier to identify if the site is methylated based on the preceding extracted features. Specifically, RAMPred [14] adopts the support vector machine(SVM) to predict the m^1^A modification site, extracting features based on nucleotide composition(NC) and nucleotide chemical properties(NCP). iRNA-3typeA [15] adopts SVM to predict m^1^A, A-to-I, and m^6^A modification sites, which extracts features based on accumulated nucleotide frequency(ANF) and NCP. iMRM [16] extracts features based on NCP, NC, Nucleotide Density(ND), Dinucleotide physicochemical properties(DPCP), and Dinucleotide Binary Encoding(DBE) and employs XGboost to predict m^1^A, m^6^A, m^5^C, ψ, and A-to-I modification sites. The above sequence features are artificially extracted, and inevitably important features of the sequences are missed due to human cognitive limitations.

Analyzing biological sequences and interpreting biological information are the key challenges in achieving biological discovery. The application of natural language processing(NLP) to sequence analysis has attracted considerable attention in processing biological sequences [17]. As biological sequences can be considered sentences, and k-mer subsequences are regarded as words [18, 19], NLP can be used to understand the structure and function encoded in these sequences [17]. Unlike traditional machine learning, deep learning (DL) methods follow an end-to-end design. Features are extracted directly based on the input sequence and the final labeling/prediction task. For example, EDLm6Apred [20] employs bidirectional long short-term memory (BiLSTM) to predict m^6^A sites, extracting features based on Word2vec, RNA word embedding [21], and one-hot encoding [22, 23]. However, LSTM, BiLSTM, and RNN cannot achieve parallel computation, leading to a long training time.

CNN can achieve parallel computation and learn local dependencies. For instance, m6A-word2vec [24] adopts CNN to identify m^6^A sites, extracting features based on Word2vec. Deeppromise [25] employs CNN to identify m^1^A and m^6^A sites, extracting features based on integrated enhanced nucleic acid composition (ENAC) [26], one-hot encoding, and RNA word embedding. However, These CNN structures only consider the contextual relationships of neighboring bases without considering the dependencies over long distances in the sequence. DeepM6ASeq [27] combines the advantages of CNN and BiLSTM by using two layers of CNN and one layer of BiLSTM to predict m^6^A sites. This approach may extract redundant features that interfere with prediction performance [28]. The attention mechanism can quantify the degree of code-to-code dependency [29]. Therefore, the application of the attention mechanism can capture the focused codes that affect the classification results. Plant6mA [30] utilizes a Transformer encoder to determine whether the input sequence contains an m6A site. However, due to the unique feature representation of transformers, these networks are primarily employed at a single scale. Although a single-scale self-attentive mechanism can focus on essential features of sequence context, it lacks information interaction with sequences at different scales. It isn't easy to learn complex word context relationships.

At present, most prediction model studies focus only on a single methylation modification, and few share the same binary classification model framework to achieve different methylation modification predictions. Even fewer cross-modification validation studies have been performed with different methylation test sets and trained models. Accounting for potential interactions between various RNA modifications, it would be interesting to use the same model to conduct cross-modification validation studies across different methylation test sets.

We present the Multi-scale Self- and Cross-attention Network (MSCAN), a novel approach designed to identify RNA methylation sites, addressing the challenges associated with current methods. Our model supports identifying twelve RNA modification types, including m^6^A, Ψ, m^1^A, m^6^Am, Am, Cm, m^7^G, Gm, Um, I, m^5^U, and m^5^C.

The MSCAN employs a unique multi-scale approach for analyzing RNA sequences. Specifically, we extracted the input 41-nucleotides (nt) sample sequence into multiple smaller subsequences centered around the sequence midpoint. To ensure accurate identification of methylation sites, the MSCAN analyzes these smaller subsequences at two distinct scales: 21-nt and 31-nt. This multi-scale analysis allows for a more comprehensive understanding of the RNA sequence context, ultimately leading to improved prediction performance. Secondly, word2vec was used to encode the three sets of sequences. Third, the three sets of sequences add positional information due to the correlation between nucleotide positions in the sequence. Four, the three sets of sequences were fed into the encoding module, which was constructed with a multi-scale self- and cross-attention network and a feed-forward network(FFN) to extract potential contributing features for methylation site prediction. Finally, methylation predicted probabilities were obtained through a linear layer and the sigmoid function. The findings demonstrated that the MSCAN model surpassed the performance of state-of-the-art methods, including m6A-word2vec, DeepM6ASeq, and Plant6mA in independent tests. A user-friendly web server for MSCAN is available at http://47.242.23.141/MSCAN/index.php.

## Result

### Evaluation metrics

In this study, we used eight common classification indicators to evaluate the prediction of the model, including Accuracy (Acc), Sensitivity (Sen), Precision (Pre), Matthews correlation coefficient (MCC), Specificity (Sp), and F1 score (F1). The formulas of these metrics are as follows:

1 $$Sensitivity,\;Recall = \frac{TP}{{TP + FN}}$$

2 $$Specificity = \frac{TN}{{TN + FP}}$$

3 $$Accuracy = \frac{TP + TN}{{TP + TN + FP + FN}}$$

4 $$Precision = \frac{TP}{{TP + FP}}$$

5 $$F1 \, score = 2 \times \frac{Precision \times Recall}{{Precision + Recall}}$$

6 $$MCC = \frac{TP \times TN - FP \times FN}{{\sqrt {(TP + FP) \times (TP + FN) \times (TN + FP) \times (TN + FN)} }}$$

Here, the true positive, true negative, false positive, and false negative are represented as TP, TN, FP, and FN, respectively. Moreover, the area under the receiver operating characteristic (AUROC) and the area under the precision-recall curve (AUPRC) are used to visually evaluate the model's overall performance.

### Results analysis

MSCAN completed model training and experimental parameter optimization based on the dataset of Chen et al. [25]. Subsequently, MSCAN completed the model's generalization ability evaluation based on the dataset of Song et al. [5]. Specifically, based on the dataset of Chen et al., this paper first compared the performance of MSCAN with different combinations of input sequences on the training data. Second, the performance of MSCAN with different feature encoding was compared. Third, we compared the performance of different MSCAN model variants. Fourth, the MSCAN was compared with state-of-the-art models based on the training data and the independent dataset of Chen et al. Fifth, the statistical significance of AUPRC values between the four models is compared. Sixth, MSCAN completed a generalization ability evaluation based on the dataset of Song et al., and MSCAN outperformed the state-of-the-art predictors for twelve modification sites. Finally, We designed a cross-modification validation experiment in which twelve models with different methylation types were compared for prediction performance based on twelve test sets, respectively. Our experiments were conducted with two Intel(R) 5218 CPUs, two RTX2080Ti GPUs, and Pytorch version 1.4.0+cu92.

Based on the training data of Chen et al., we first tried optimizing the input sequences' length according to AUPRC on the training data. Using the Word2vec embedding, we evaluated the Transformer model with 11-nt, 21-nt, 31-nt, 41-nt,51-nt,61-nt,71-nt,81-nt,91-nt,and 101-nt RNA sequences as the input on the five-fold cross-validation [5, 25]. As shown in Table 1, the input of the 11-nt sequence obtained the worst performance. The reason may be that too few bases in the 11-nt sequence affect feature extraction. The input of the 41-bp sequence obtained the best average performance of all the modifications, It may be worth mentioning that the 41-nt of the input sequence is also optimal for the XGboost and SVM method [14, 16], so we choose 21-nt, 31-nt, and 41-nt RNA sequences as input sequences to achieve different combinations of input sequences.

**Table 1:** Evaluation results of five-fold cross-validation of transformer based on the training data of Chen et al.

Length(nt)	AUROC	ACC	Sen	Precision	MCC	Spe	F-1	AUPRC
11	0.8955	93.55	44.26	**77.14**	55.44	**98.65**	56.25	0.6389
21	0.9213	**95.01**	59.81	74.42	64.11	98.16	66.32	0.7189
31	0.9361	93.55	64.80	66.94	62.31	96.61	65.85	0.7390
41	**0.9484**	93.48	**74.36**	61.27	63.97	95.37	67.18	**0.7469**
51	0.9401	94.63	61.54	74.23	64.73	97.89	67.29	0.7401
61	0.9430	94.56	67.27	67.89	64.61	97.07	67.58	0.7078
71	0.9249	93.64	67.21	65.60	62.89	96.36	66.40	0.7272
81	0.9440	94.94	70.00	66.04	**65.25**	97.01	**67.96**	0.7170
91	0.9327	94.93	60.19	73.86	64.01	98.08	66.33	0.7119
101	0.9230	93.33	70.94	61.03	62.16	95.53	65.61	0.7449

Bold indicates the best performance

The combination of input sequences with different scale order is an important parameter that affects the performance of the training model. The performance of the MSCAN model with the different combinations of input sequences on the training data is shown in Table 2. MSCAN shows the best prediction performance when the combination is “21-nt + 41-nt + 31-nt”. According to the MSCAN model design, “21-nt + 41-nt + 31-nt” input sequences are entered into the model to implement three attention mechanisms, including the self-attention calculation mechanism for the 21-nt sequence, and the cross-attention calculation mechanisms for both “21-nt + 41-nt” and “21-nt + 31-nt” combinatorial sequences.

**Table 2:** Evaluation results of MSCAN on five-fold cross-validation with different input sequences based on the training data of Chen et al.

combinations of sequences(nt)	AUROC	ACC	Sen	Precision	MCC	Spe	F-1	AUPRC
21 + 31 + 41	0.9491	**95.24**	58.59	73.42	63.11	98.26	65.17	0.7695
21 + 41 + 31	**0.9618**	94.86	59.69	**83.70**	**68.11**	**98.72**	69.68	**0.7949**
31 + 21 + 41	0.9257	94.63	55.93	78.57	63.59	98.48	65.34	0.7419
31 + 41 + 21	0.9463	95.09	**68.47**	72.38	67.73	97.57	**70.37**	0.7632
41 + 21 + 31	0.9318	94.55	63.87	73.08	65.38	97.64	68.17	0.7617
41 + 31 + 21	0.9427	93.86	65.41	71.90	65.20	97.10	68.50	0.7642

Bold indicates the best performance

### Comparison analysis of different feature encoding methods

**Fig. 1:**
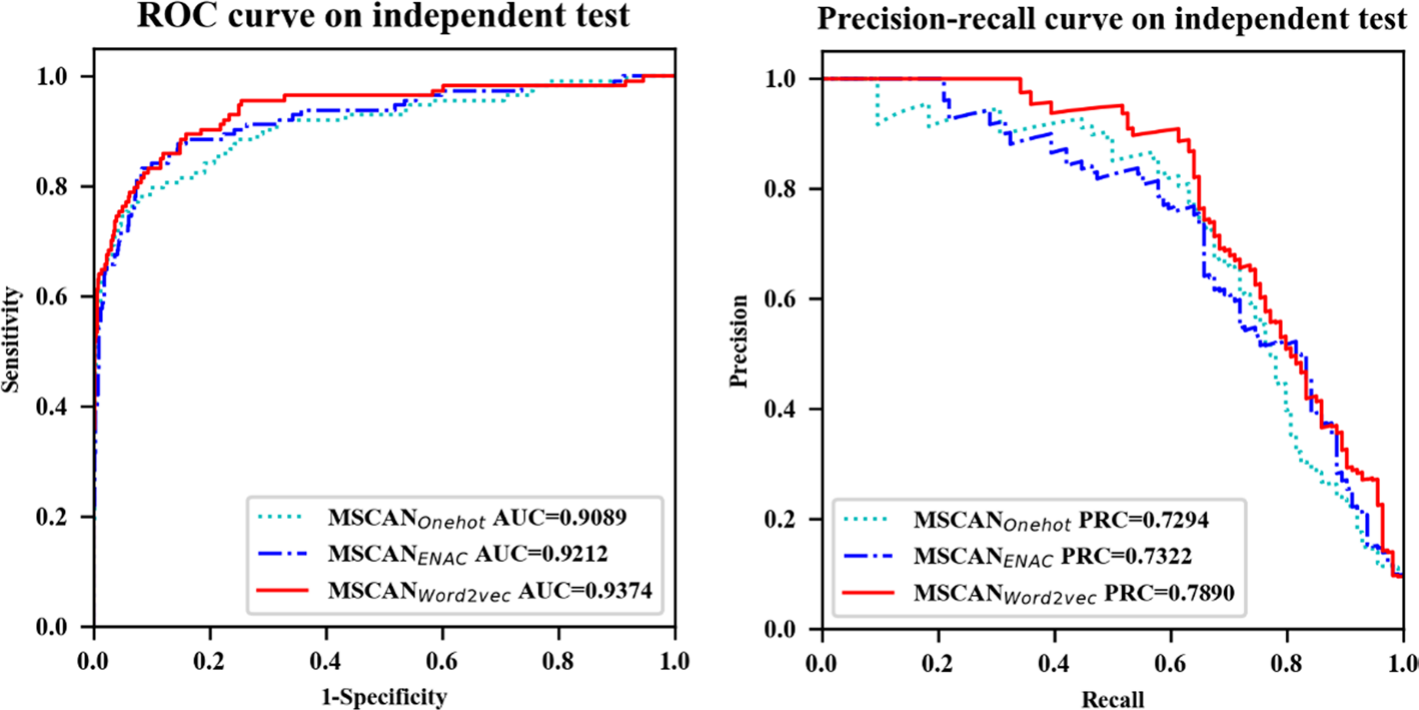
Performance of the MSCAN model based on the different feature encoding

In this section, we evaluate the performance of three distinct feature encoding methods—Word2vec, One-hot, and ENAC—utilizing the same MSCAN model for predicting m^1^A sites on the test data of Chen et al. The outcomes of this comparison are presented in Fig. 1 and Table 3, demonstrating that Word2vec consistently surpasses the other two encoding methods across all performance indices.

**Table 3:** MSCAN model evaluation results with different feature encodings based on the test data of Chen et al.

Encoding	AUROC	ACC	Sen	Precision	MCC	Spe	F-1	AUPRC
One-hot	0.9089	95.13	55.26	86.30	66.77	99.12	67.38	0.7294
ENAC	0.9212	94.81	55.26	81.82	64.71	98.77	65.97	0.7322
Word2vec	**0.9374**	**95.69**	**58.77**	**90.54**	**70.95**	**99.39**	**71.28**	**0.7890**

Bold Indicates the best performance

The superior performance of Word2vec can be attributed to the limitations of the One-hot and ENAC encoding methods. While One-hot encoding focuses on the local information of individual bases, and ENAC encoding considers both nucleic acid composition and position information, both methods neglect the semantic information inherent in the sequence context. In contrast, Word2vec prioritizes the contextual relationships between bases, resulting in a more effective representation of the sequence.

Our findings highlight the importance of selecting appropriate feature encoding methods for improved prediction accuracy, with Word2vec emerging as a particularly advantageous choice for the MSCAN model in the context of RNA methylation site prediction.

### Comparison with different variants of the MSCAN model

**Fig. 2:**
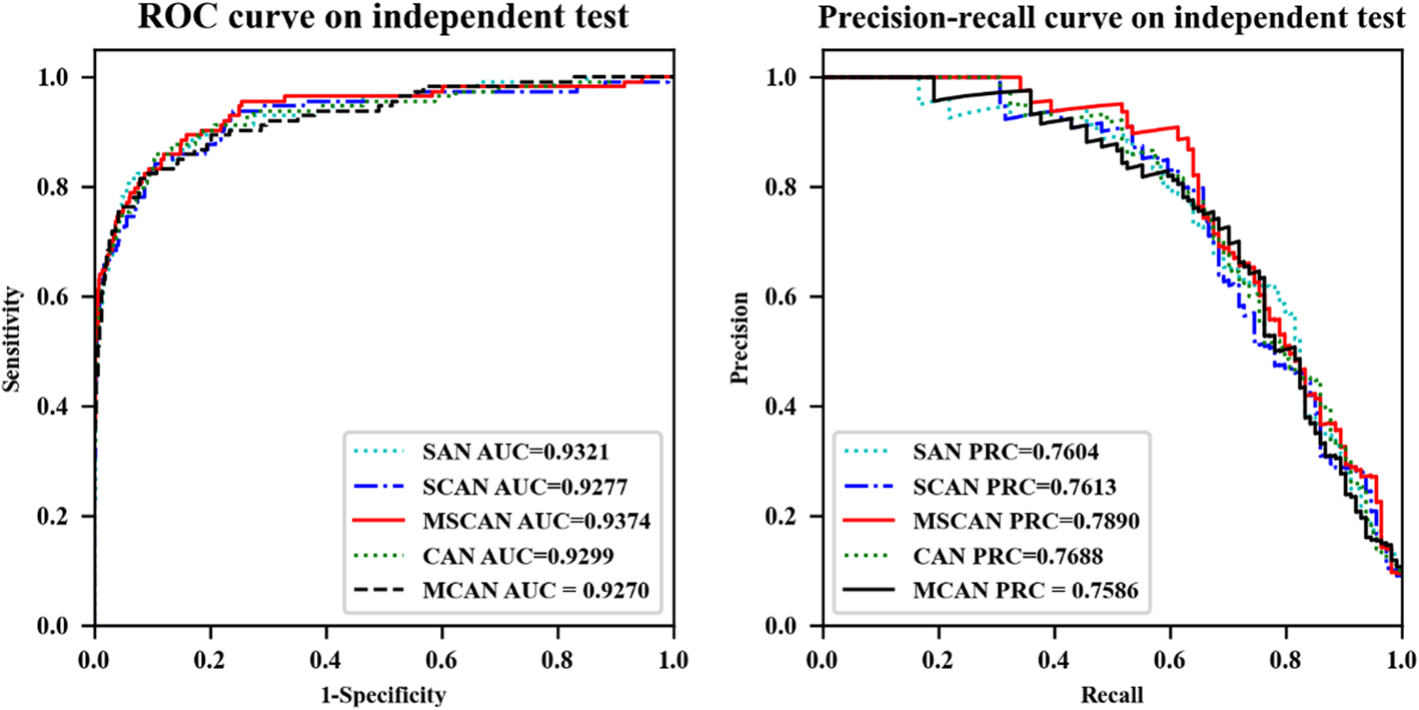
Performance of MSCAN and variant model on the test data

We conducted ablation experiments to assess the contribution of key components within our proposed MSCAN model based on the test data of Chen et al. Utilizing Word2vec for RNA sequence encoding, we constructed four sub-networks: self- and cross-attention network (SCAN), self-attention network (SAN), multi-scale cross-attention network (MCAN), and cross-attention network (CAN). SCAN represents MSCAN with one cross-attention module removed, SAN is SCAN devoid of cross-attention, MCAN is MSCAN without self-attention, and CAN is MCAN with one cross-attention module removed. The outcomes of these experiments are depicted in Fig. 2 and summarized in Table 4.

**Table 4:** Comparing MSCAN and variant model evaluation results based on test data of Chen et al.

Classifiers	AUROC	ACC	Sen	Precision	MCC	Spe	F-1	AUPRC
SAN	0.9321	95.05	55.26	85.14	66.24	99.04	67.02	0.7604
SCAN	0.9277	95.29	53.51	**91.04**	67.73	**99.47**	67.40	0.7613
MSCAN	**0.9374**	**95.69**	**58.77**	90.54	**70.95**	99.39	**71.28**	**0.7890**
CAN	0.9299	94.89	52.63	85.71	64.81	99.12	65.21	0.7688
MCAN	0.9270	94.81	55.26	81.82	64.71	98.77	65.97	0.7586

Bold Indicates the best performance

SAN contains only self-attention; SCAN, and MSCAN are combinations of self- and cross-attention; CAN and MCAN are combinations of only cross-attention

SAN serves as the baseline model in this comparison. Upon the integration of cross-attention modules, the area under the precision-recall curve (AUPRC) for SCAN and MSCAN models increased by 0.09% and 2.86%, respectively. These results highlight the importance of incorporating cross-attention mechanisms within the MSCAN model for improved performance in predicting RNA methylation sites. Consequently, our findings emphasize the value of the multi-scale self- and cross-attention approach employed by MSCAN in advancing the understanding of RNA modifications and their functional implications.

### Comparison with state-of-the-art approaches

**Fig. 3:**
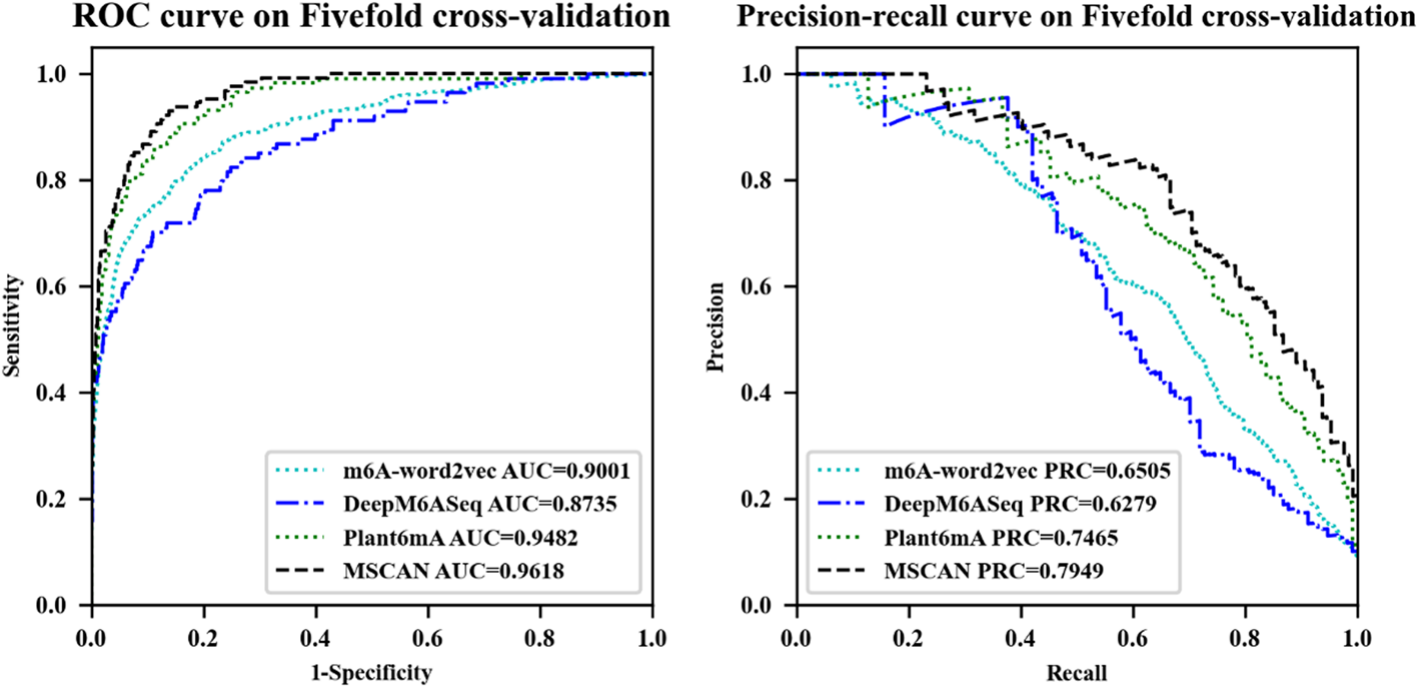
Performance of the different models on the training data

We compared MSCAN with several state-of-the-art models, including m6A-word2vec, DeepM6ASeq, and Plant6mA. To ensure robust evaluation, we employed a fivefold cross-validation on the training data of Chen et al. As shown in Fig. 3 and Table 5, Our results demonstrate that MSCAN outperforms the other models, substantially improving prediction accuracy.

**Table 5:** Evaluation results of MSCAN and other state-of-the-art models based on five-fold cross-validation using the training data of Chen et al.

Classifiers	AUROC	ACC	Sen	Precision	MCC	Spe	F-1	AUPRC
m6A-word2vec	0.9001	93.30	53.79	66.18	56.10	97.25	59.35	0.6505
DeepM6ASeq	0.8735	93.38	46.49	70.67	54.02	98.07	56.08	0.6279
Plant6mA	0.9482	93.48	**74.36**	61.27	63.97	95.37	67.18	0.7465
MSCAN	**0.9618**	**94.86**	59.69	**83.70**	**68.11**	**98.72**	**69.68**	**0.7949**

Bold indicates the best performance

In particular, MSCAN achieves a 4.84% enhancement in the AUPRC metric compared to the second-best performing model, Plant6mA. This superior performance can be attributed to utilizing the multi-scale self- and cross-attention mechanisms in MSCAN, as opposed to the self-attention mechanism employed by Plant6mA. The results underscore the effectiveness of MSCAN in identifying RNA methylation sites.

**Fig. 4:**
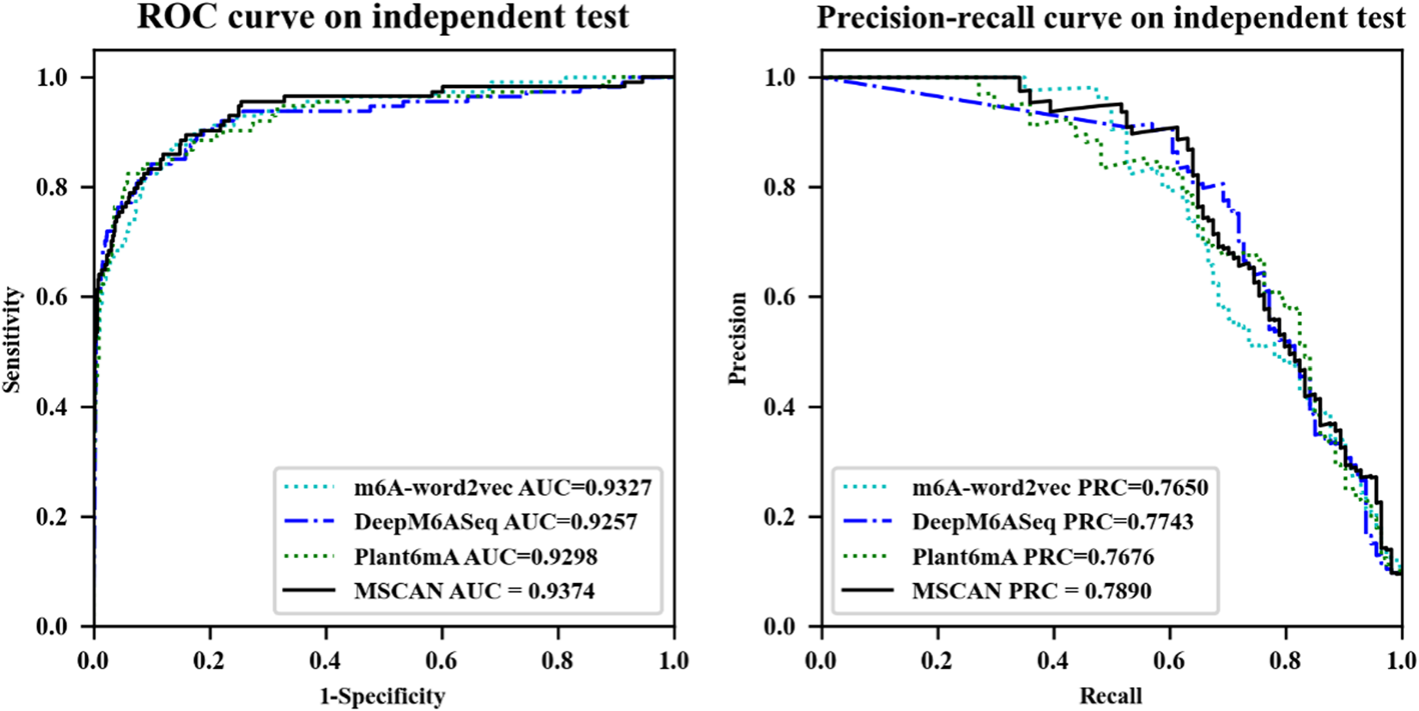
The ROC and PRC of MSCAN and other state-of-the-art models on the test data

Next, we compare the performance of MSCAN with other state-of-the-art models using the test data of Chen et al. The results, as illustrated in Fig. 4 and summarized in Table 6, demonstrate the superior performance of MSCAN in predicting RNA methylation sites.

**Table 6:** Evaluation results of MSCAN and other state-of-the-art models based on the test data of Chen et al.

Classifiers	AUROC	ACC	Sen	Precision	MCC	Spe	F-1	AUPRC
m6A-word2vec	0.9327	93.62	**67.54**	64.17	62.32	90.43	65.81	0.7650
DeepM6ASeq	0.9257	95.37	65.79	79.79	70.00	98.33	**72.12**	0.7743
Plant6mA	0.9298	95.13	56.14	85.33	66.89	99.04	67.72	0.7676
MSCAN	**0.9374**	**95.69**	58.77	**90.54**	**70.95**	**99.39**	71.28	**0.7890**

Bold indicates the best performance

MSCAN outperforms DeepM6ASeq and m6A-word2vec by 1.47% and 2.4% in terms of AUPRC, respectively. This enhanced performance can be attributed to the multi-scale self- and cross-attention network's ability to capture meaningful sequence encodings for more accurate classification. Furthermore, MSCAN surpasses Plant6mA by 2.14% in AUPRC, which may further verify the limitations of the single-scale self-attention mechanism in learning complex contextual relationships between sequence elements. The integration of the cross-attention mechanism enables the model to discern deeper sequence meanings, thus improving its performance.

### Assessing model reliability

To evaluate the reliability of our proposed model, we performed one hundred replications of experiments using the test data from Chen et al., evaluating the m6A-word2vec, DeepM6ASeq, Plant6mA, and MSCAN models. In each replication, we used the same test data and ran each model under identical conditions to ensure experimental consistency.

To evaluate the statistical significance of AUPRC values between different methods, we employed Student's t-test [31]. This statistical method helps determine whether performance differences between different methods are significant. Table 7 below shows the *p* values for the difference in the performance of the four classifiers.

**Table 7:** A statistically significant correlation matrix for the difference in the performance of the four classifiers

Classifiers	Classifiers			
	m6A-word2vec	DeepM6ASeq	Plant6mA	MSCAN
m6A-word2vec				
DeepM6ASeq	0.001243			
Plant6mA	2.905E−44	8.01217E−35		
MSCAN	4.71415E−64	1.25245E−58	0	

### Assessing model generalization ability

Based on the data set of Song et al., the generalization ability of MSCAN was evaluated by training the model individually for each methylation type. As presented in Table 8, the MSCAN model consistently outperforms state-of-the-art models, including m6A-word2vec, DeepM6ASeq, and Plant6mA. This result provides empirical evidence of the model's generalizability across diverse methylation site prediction tasks.

**Table 8:** Compare MSCAN to other methods under AUC

Classifiers	m^6^A	Ψ	m^1^A	m^6^Am	Am	Cm	Gm	Um	m^5^C	m^7^G	m^5^U	I
m6A-word2vec	0.9773	0.7060	0.8385	0.9867	0.9174	0.9120	0.9554	0.8467	0.9611	0.7505	0.9499	0.6180
DeepM6ASeq	0.9752	0.7510	0.8289	0.9837	0.9213	0.9173	0.9538	0.8716	0.9675	0.7527	0.9584	0.5872
Plant6mA	0.5964	0.5478	0.7268	0.7826	0.8110	0.8016	0.8279	0.7847	0.6806	0.6690	0.9528	0.5137
MSCAN	**0.9834**	**0.7622**	**0.8541**	**0.9904**	**0.9292**	**0.9203**	**0.9577**	**0.8966**	**0.9729**	**0.7994**	**0.9674**	**0.6569**

Bold indicates the best performance

Theoretically, the self- and cross-attention mechanism employed by the MSCAN model enables it to capture long-range dependencies and complex interactions between input features more effectively than other models, such as Recurrent Neural Networks (RNNs) and Convolutional Neural Networks (CNNs). This characteristic is particularly advantageous in discerning biologically relevant patterns in methylation site prediction, which may contribute to the model's enhanced generalizability.

### Comparison with cross-modification validation approaches

Thus far, our results have demonstrated the model's robust classification performance. Notably, a significant advantage of the proposed MSCAN model is its ability to learn the underlying associations among different RNA modifications. Previous studies have revealed clear evolutionary and functional cross-talk among various post-translational modifications of proteins [32] and histone and chromatin modifications [33]. Such associations might also exist at the epi-transcriptome level among different RNA modifications.

**Fig. 5:**
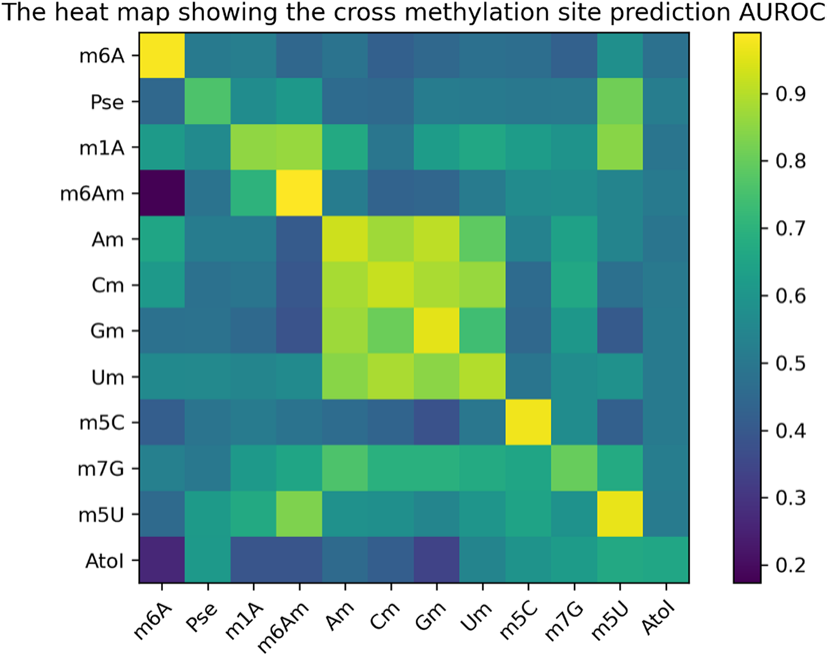
Heat map of different AUROC values in cross-methylation validation. The horizontal axis is the model type, and the vertical axis is the test data type

To better understand the inherent shared structures among different RNA modifications, we performed cross-modification validation on the second dataset. The resulting AUROC values are displayed in Fig. 5. As the figure shows, cross-modification validation yielded poorer prediction results than those obtained using modification-consistent data and models, indicating the specificity of our method for a particular modification.

Interestingly, in experiments where the test dataset and model were inconsistent, some groups achieved high AUROC values greater than 0.85, suggesting strong and significant positive associations among certain RNA modifications, even those originating from different nucleotides. This observation implies the existence of regions intensively modified by multiple RNA modifications, which likely serve as key regulatory components for the epi-transcriptome layer of gene regulation. Notably, the sequence signatures of these key regulatory regions are largely shared among different RNA modifications (including those that modify different nucleotides) and were successfully captured by our model. As presented in Table 9, the most strongly associated modifications originated from the same type of base, with A and G belonging to purine-like bases, and C and U belonging to pyrimidine bases.

**Table 9:** Association of RNA modifications revealed by MSCAN

Dataset	Model	AUROC	AUPRC	ACC	Dataset	Model	AUROC	AUPRC	ACC
Am	Am	0.9292	0.9231	86.36	Gm	Gm	0.9577	0.9637	88.33
Gm	Am	0.9082	0.9233	81.11	Am	Gm	0.8695	0.8809	80.16
Cm	Am	0.8724	0.8906	73.51	Cm	Gm	0.8083	0.8280	72.18
Um	Am	0.7884	0.7839	60.00	Um	Gm	0.7387	0.7659	66.34
Cm	Cm	0.9203	0.9273	83.44	Um	Um	0.8966	0.8756	82.92
Um	Cm	0.8647	0.8514	79.75	Cm	Um	0.8859	0.8739	81.78
Am	Cm	0.8830	0.8862	72.31	Am	Um	0.8458	0.8282	78.51
Gm	Cm	0.8865	0.9070	69.44	Gm	Um	0.8480	0.8589	76.66

To further verify this finding, we compared Am, Gm, Cm, and Um correlations through local BLAST [34] software. First, the Am, Gm, and Cm comparison libraries are established based on the Am data set, Gm data set, and Cm data set respectively. Secondly, the Am, Gm, Cm, and Um data sets are used to compare the comparison libraries with different methylation in pairs. Then, the BLAST output table is obtained. Finally, compare the average value of the comparison result "bit-score". As shown in Table 10, the average bit-score value of the Gm sequence compared to the Am comparison library is high, indicating that the Am sequence and the Gm sequence are highly similar. Similarly, the average bit-score value of the Um sequence compared to the Cm comparison library is high, indicating that the Um sequence and Cm sequence similarity is high, which may validate the idea that the most closely related modifications originate from the same type of bases.

**Table 10:** Compare the average bit-score of various methylated sequences

Query subject	Am	Gm	Cm	Um
Am		9.679614	5.866832	3.30773
Gm			3.357072	1.639136
Cm				8.793488
Um				

Our model provides experimental verification of the existence of an inherent shared structure between different RNA modifications. These findings underscore the potential of the MSCAN model in advancing our understanding of the complex interplay between various RNA modifications and their functional implications.

### Web server

**Fig. 6:**
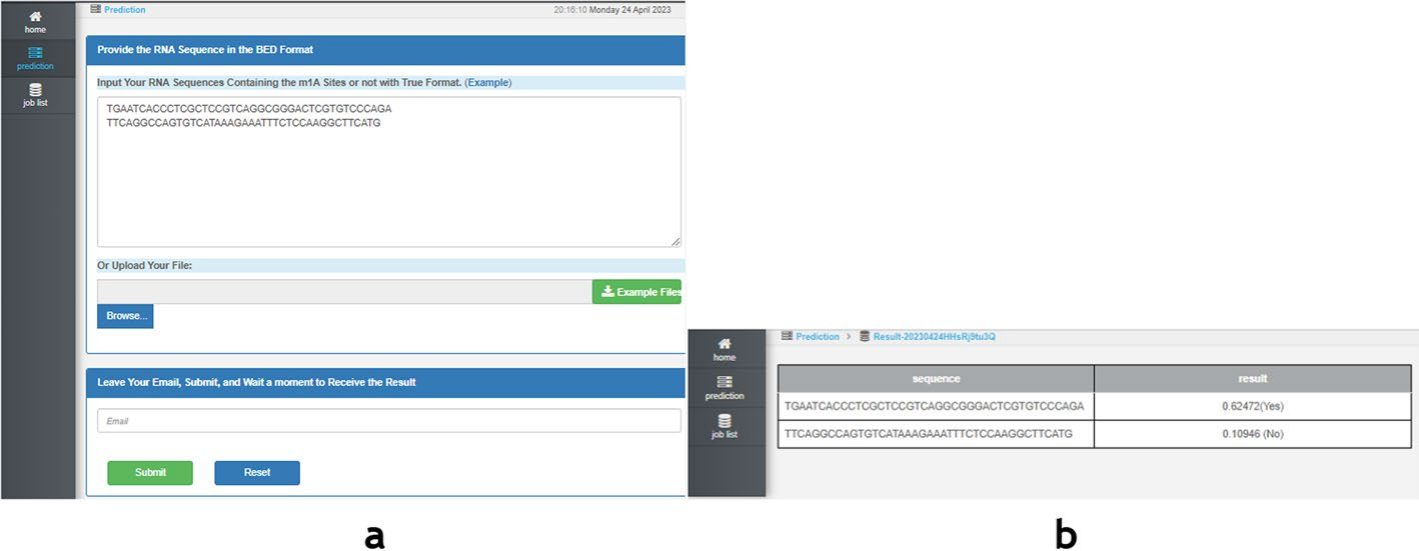
Webserver interface. a. Input interface. b. Prediction result

We have developed a user-friendly web server for predicting twelve widely occurring human RNA modification sites (m^6^A, m^1^A, m^5^C, m^5^U, m^6^Am, m^7^G, Ψ, I, Am, Cm, Gm, and Um), accessible at http://47.242.23.141/MSCAN/index.php, to facilitate the use of the MSCAN model for RNA methylation site prediction. Take the step of predicting the m^1^A methylation site as an example. First, click the “Prediction” button and select the “m^1^A” successively. Next, type or paste the RNA sequence, as shown in Fig. 6a. Third, leave your email address in the input box and click the “submit” button. After a calculation period, the prediction results will be displayed in a table, as shown in Fig. 6b. This intuitive web server offers researchers an efficient and convenient platform for employing the MSCAN model in their investigations of RNA modifications and their functional implications.

## Discussion

First, based on the test data of Chen et al., we compared the performance of various features based on the MSCAN model, including One-hot encoding, ENAC, and Word2vec. The results reveal that Word2vec outperforms One-hot and ENAC in predicting AUROC and AUPRC. Specifically, the AUPRC of MSCAN_word2vec_ is 5.96% and 5.68% higher than that of MSCAN_One-hot_ and MSCAN_ENAC_, respectively. These findings are in line with Zhang et al.'s study [20], which highlights that One-hot focuses on local semantic information while ENAC only considers the sequence's nucleic acid composition and position, neglecting more profound semantic information. Conversely, Word2vec captures the contextual semantic information of the sequence, significantly enhancing the model's predictive capability.

Second, based on the test data of Chen et al., we assessed the impact of various MSCAN components by comparing the performance of different MSCAN variants, such as SCAN, SAN, MCAN, and CAN. Experimental results show that MSCAN reduces AUPRC by 3.04% and 2.77% respectively after deleting a self-attention module or a cross-attention module. This finding is consistent with Sun et al.'s study [35], which posits that the removal of self- or cross-attention modules leads to diminished model performance. When both the Multi-Scale and cross-attention modules are removed, the AUPRC of MSCAN decreases by 2.86%. This result aligns with Chen et al.'s study [36], which emphasizes that cross-attention effectively learns multi-scale transformer features for data recognition.

Third, we compared the performance of m6A-word2vec, DeepM6ASeq, Plant6mA, and MSCAN based on the test data of Chen et al. MSCAN's AUROC and AUPRC outperformed the other three state-of-the-art models. In particular, MSCAN surpassed Plant6mA by 2.14% in terms of AUPRC. This study substantiates that the utilization of multi-scale input and cross-attention allows the model to extract diverse features and provide deep semantics, which Plant6mA cannot achieve through information fusion from multiple scales. This conclusion is supported by Guo et al.'s study [37], which demonstrated that multi-scale transformers could extract rich and robust features from different scale inputs.

Four, To make fair comparisons with m6A-word2vec, DeepM6ASeq, Plant6mA methods, we tested MSCAN on twelve RNA modification datasets(m6A, m1A, m5C, m5U, m6Am, m7G, Ψ, I, Am, Cm, Gm, and Um). The results show that MSCAN outperforms all other competing methods. our predicted results may also be consistent with biological insights, which illustrates that MSCAN has good robustness.

Five, based on the dataset of Song et al., we designed a cross-modification validation experiment in which twelve different methylation models were tested using twelve sets of methylation test datasets, respectively. We discovered that the most strongly associated modifications originated from the same base class, such as A and G belonging to purine-like bases. The AUROC and AUPRC metrics of the Am test set on the Gm prediction model are second only to the Am test set on the similar Am prediction model. This finding is consistent with Song et al.'s study [5], which proposed the existence of an inherent shared structure between different RNA modifications.

Lastly, we compared Am, Gm, Cm, and Um correlations through local BLAST software. We found the average bit-score value of the Gm sequence compared to the Am comparison library is high, indicating that the Am sequence and the Gm sequence are highly similar. Similarly, the average bit-score value of the Um sequence compared to the Cm comparison library is high, indicating that the Um sequence and Cm sequence similarity is high, which may validate the idea that the most closely related modifications originate from the same type of bases.These findings underscore the potential of the MSCAN model in advancing our understanding of the complex interplay between some RNA modifications and their functional implications.

## Conclusions

This study presents a novel multi-scale cross-attention network (MSCAN) for predicting RNA methylation sites. By combining multi-scale, self-, and cross-attention mechanisms, MSCAN effectively extracts in-depth features from 41 base pair sequences at various scales. The model outperforms state-of-the-art predictors for all twelve modification sites, demonstrating its strong generalization ability.

Crucially, through the cross-modification validation experiments, our model unveils significant associations among different types of RNA modifications in terms of their related sequence contexts. This finding offers valuable insights into the complex relationships between RNA modifications and their respective sequence environments.

It is worth noting that the data set samples of the MSCAN model have the following conditions: (1) The sample is a 41-nt fixed-length sequence, (2) The methylation site must be in the center of the sequence, (3) The sample sequence must have a label. It may seem that MSCAN may only be tested by this method.We hope that in the future, targeting the characteristics of RNA sequences of different lengths, the model structure is adjusted to better capture and utilize these characteristics, and focusing particularly on studies that investigate the biological functions and regulatory mechanisms of different RNA sequence lengths.

## Materials and methods

### Datasets

**Fig. 7:**
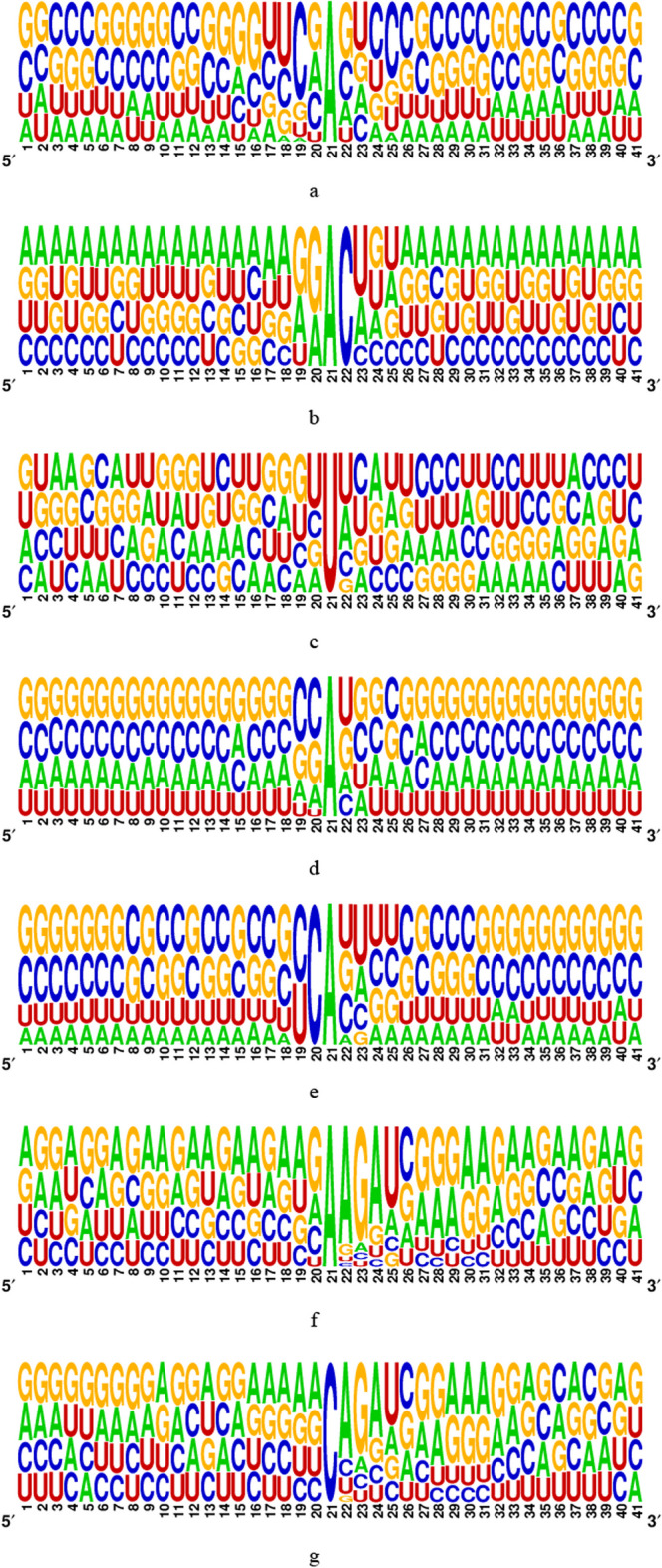

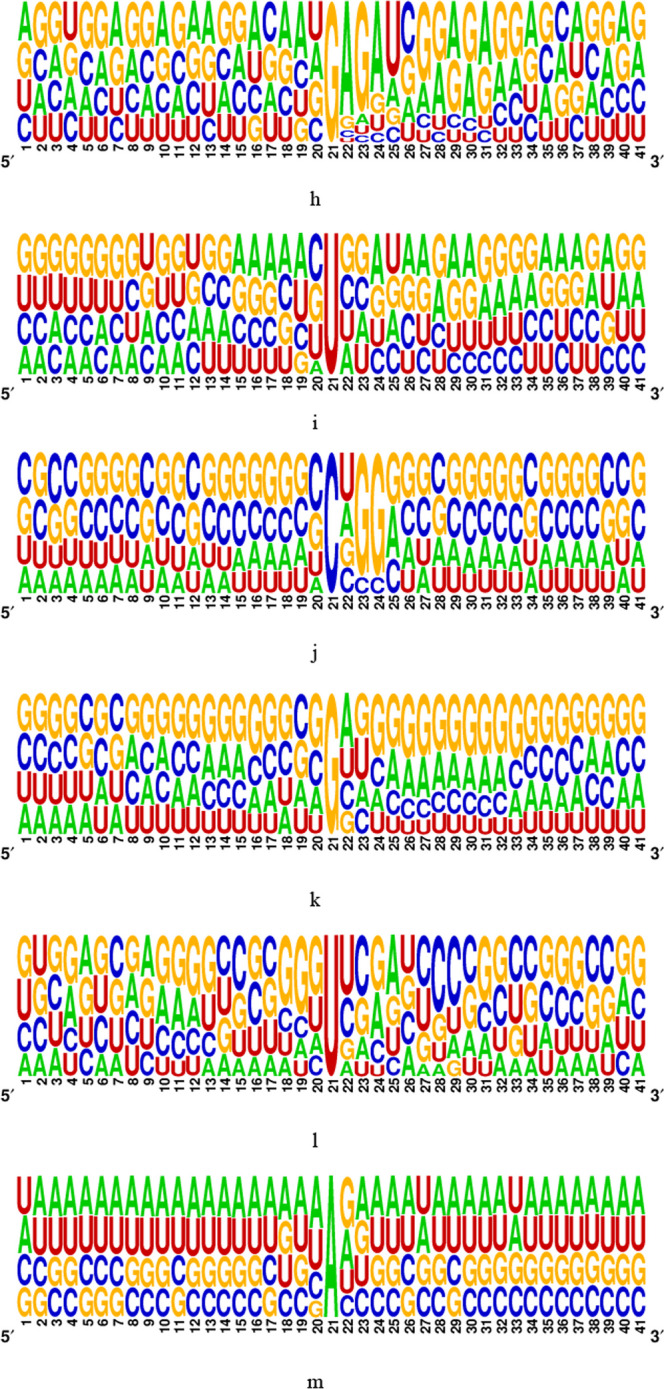
The motif of methylation sites. **a** m^1^A in the dataset of Chen et al. **b** m^6^A. **c** Ψ. **d** m^1^A. **e** m^6^Am. **f** Am. **g** Cm. **h** Gm. **i** Um. **j** m^5^C. **k** m^5^U. **l** m^7^G. **m** I in the dataset of Song et al.

In the present study, the benchmark datasets employed to train and test the proposed methods were gathered from previous works [5, 25]. These datasets encompass twelve distinct types of RNA modifications, namely m^6^A, m^1^A, m^5^C, m^5^U, m^6^Am, m^7^G, Ψ, I, Am, Cm, Gm, and Um from H. sapiens. They can be downloaded from http://47.242.23.141/MSCAN/index.php, and detailed information is provided in Table 11. To maintain consistency, all sequence samples were adjusted to a length of 41-nt, with the modified or unmodified site positioned at the center. In cases where the original sequence length fell short of 41-nt, we employed a padding technique, appending “−” to the head or tail of the sequence, to ensure a uniform length of 41-nt across all samples. The raw RNA datasets are represented as $R_{0} = \left\{ {x^{n} } \right\}_{n = 1}^{N}$, where *N* is the sequence number, and each $x^{n} \in {\mathbb{R}}^{L}$ is an RNA sequence. Each entry $x_{i}^{n} \in \left\{ {A,C,G,U,` - ^{\prime}} \right\}\;or\;x_{i}^{n} \in \left\{ {A,C,G,U} \right\},i = 1,2,3, \ldots ,L$, where *L* is the fixed sequence length. The model training and experimental parameter optimization of MSCAN are based on the dataset of Chen et al., and the evaluation of MSCAN generalization capability is based on the dataset of Song et al. The ratio of positive-to-negative samples of Chen's and Song's datasets was 1:10 and 1:1, respectively, as shown in Table 11. The corresponding sequences were followed by aligning of the sequences according to sequence-logo representations rendered using the WebLogo program [38, 39], As shown in Fig. 7.

**Table 11:** A statistic of the training and test datasets

Full name	Dataset	Original base	Number of positive	Number of negative	Source of data
1-Methyladenosine	m^1^A_train0	A	593	5930	Chen et al.[25]
	m^1^A_test0	A	114	1140	
N6-methyladenosine	m^6^A_ train	A	41,307	41,307	Song et al.[5]
	m^6^A_test	A	5901	5901	
1-Methyladenosine	m^1^A_train	A	7357	7357	
	m^1^A_test	A	1051	1051	
5-Methylcytidine	m^5^C_train	C	5953	5953	
	m^5^C_test	C	850	850	
5-Methyluridine	m^5^U_train	U	863	863	
	m^5^U_test	U	123	123	
N6,2′-*O*-dimethyl adenosine	m^6^Am_train	A	1172	1172	
	m^6^Am_test	A	167	167	
7-Methylguanosine	m^7^G_train	G	605	605	
	m^7^G_test	G	86	86	
Pseudouridine	Pse_train	U	1989	1989	
	Pse_test	U	284	284	
2′-*O*-methyladenosine	Am_train	A	848	848	
	Am_test	A	121	121	
2′-*O*-methylcytidine	Cm_train	C	1058	1058	
	Cm_test	C	151	151	
2′-*O*-methylguanosine	Gm_train	G	636	636	
	Gm_test	G	90	90	
2′-*O*-methyluridine	Um_train	U	1438	1438	
	Um_test	U	205	205	
Inosine	I_train	A	5164	5164	
	I_test	A	737	737

### Feature encoding representation

Achieving an effective feature encoding representation of the sequence is crucial for improving the evaluation metrics of a model. This study uses Word2vec to transform the sequence into embedded vector representations. Since its introduction in 2013, Word2vec has significantly advanced the performance of a wide array of natural language processing (NLP) tasks.

The Word2vec methodology offers two different frameworks for encoding: Skip-gram and Continuous Bag of Words (CBOW). The Skip-gram approach predicts contextual information surrounding a given word, whereas the CBOW model generates an embedding for the target word based on its contextual associations. These embeddings are derived through a neural network application, adeptly capturing the inherent relationships within the data.

We developed an RNA embedding approach by treating RNA sequences as sentences and k consecutive RNA nucleotides (k-mers) as words within these sentences. Mathematically, we define the mapping from single nucleotides to the vector representation of k-mers $f:\sum\nolimits_{{}}^{L} { \mapsto Y^{L - k + 1} }$, which is subsequently fed into the neural network for training. This process results in d-dimensional embedded vectors, denoted by $X_{m}^{n} \in {\mathbb{R}}^{{m \times d_{m} }}$, where *m* = *L* − *k* + 1, and *d*_*m*_ represents the embedding dimension. Gene2vec [21] demonstrated that 3-mers provide the optimal prediction performance. Consequently, we adopted a 3-mers encoding strategy for the input data. Specifically, we employed a sliding window of size 3-nt to slide 41-nt sample sequences with one stride, generating a sequence of 39 words. Each word corresponds to an index in all possible 3-mer combinations(105 or 65 types). Given the relatively large dataset and limited word types in our corpus, we chose the Continuous Bag of Words (CBOW) model for encoding, as it offers faster training times than the Skip-gram model. We used the grid-search strategy for the optimization of the parameters for the experiments, word vector dimension in [100,3 00]. Feature encoding with a word vector dimension of 100 achieved the best performance. In summary, each 3-mer is converted into a word vector, transforming a 41-nt sequence into a 39 × 100 matrix, where 100 represents the word vector dimension.

### Model

**Fig. 8:**
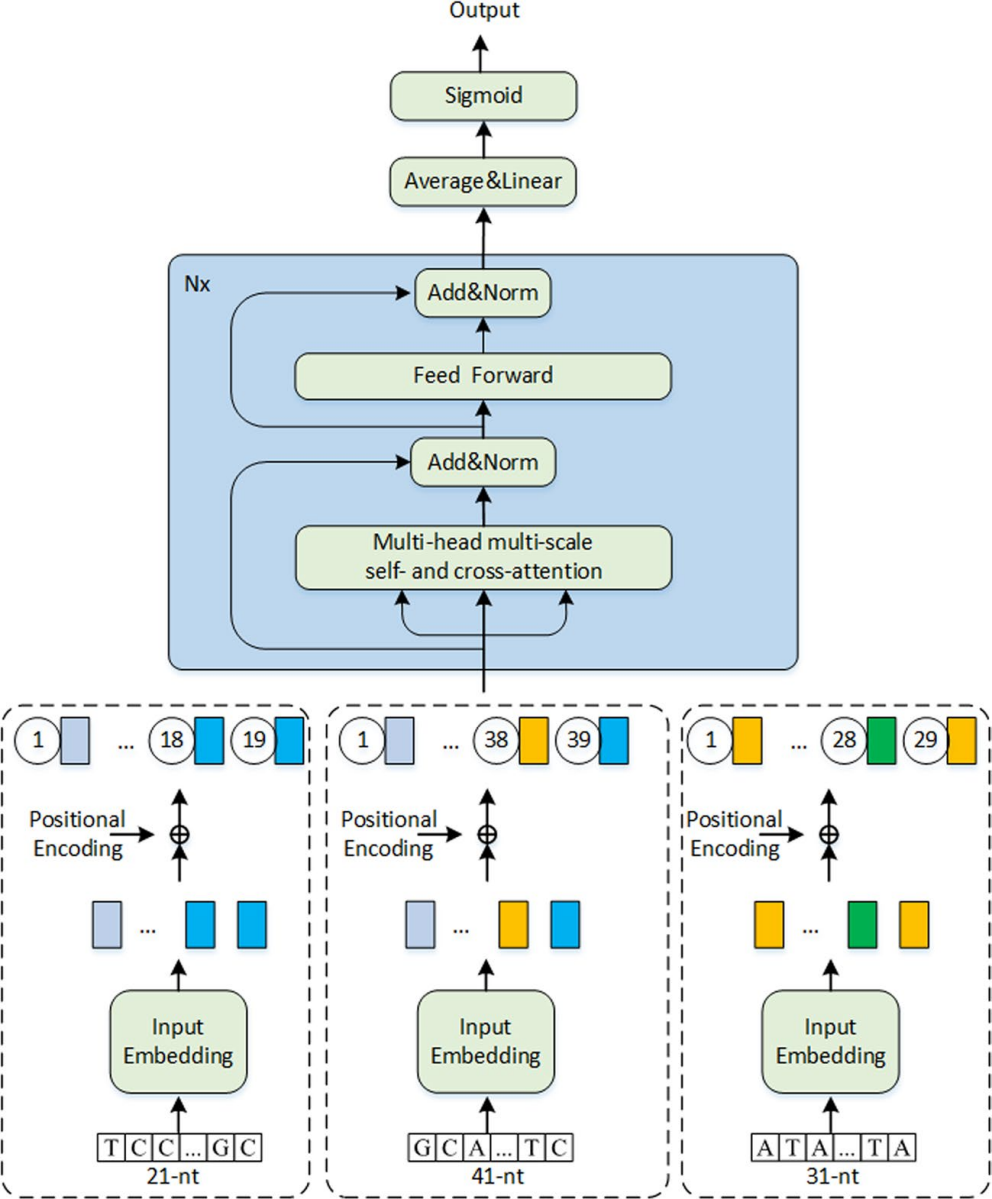
Structure of our computational framework based on multi-scale self- and cross-attention network to predict m^1^A methylation site

As shown in Fig. 8, MSCAN represents an innovative DL architecture that employs a combination of multi-scale self- and cross-attention mechanisms and point-wise, fully connected layers in the encoder. This innovative approach enables the effective modeling of both intra- and inter-sequence interactions across a wide range of scales within RNA-seq data by transforming local RNA sequences into high-dimensional vectors via representations through its multi-scale self- and cross-attention networks. MSCAN efficiently extracts crucial RNA sequence features, thereby facilitating the accurate prediction of m^1^A modifications.

**Fig. 9:**
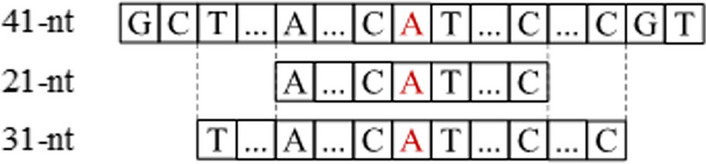
Schematic diagram of the obtained subsequences

The results of this study indicate that the nucleotide base neighboring the methylation site is instrumental in determining the specific type of methylation site and its potential functional consequences [40–42]. Therefore, the original sample sequence was extracted with two subsequences. These subsequences were centered on the sequence midpoint. One subsequence was 21-nt long, and the other was 31-nt long, as shown in Fig. 9.

In this paper, we represent the dataset as a collection of sample sequences, each consisting of a main sequence and two subsequences. The dataset can be expressed as  ${\{({\text{x}}}_{s0}^{1}, {\text{x}}_{s1}^{1} ,{\text{x}}_{s2}^{1} ,{\text{y}}^{1} )$, $({\text{x}}_{s0}^{2}, {\text{x}}_{s1}^{2}, {\text{x}}_{s2}^{2}, {\text{y}}^{2} )$, ⋯, ${\text{(x}}_{s0}^{n} , {\text{x}}_{s1}^{n}, {\text{x}}_{s2}^{n} ,{\text{y}}^{n} )\}$, where $y^{n} \in \left\{ {0,1} \right\}$, $x_{s0}^{i} ,x_{s1}^{i} ,x_{s2}^{i}$ are the three sequences of the i-th sample, $x_{s0}^{i}$ is the main sequence, with s0 = 41, $x_{s1}^{i} ,x_{s2}^{i}$ is the subsequence, with s1 = 21, and s2 = 31. Experiments show that the performance of trained models exhibits variability when the order of input sample sequences is altered, as shown in Table 1. MSCAN employs the Word2vec encoder to encode word vectors for these sequences. For example, sequences with lengths 21-nt, 41-nt, and 31-nt are transformed into three distinct matrices of varying dimensions: 19 × 100, 39 × 100, and 29 × 100, respectively.

To account for the lack of recursion or convolution in the model, it is necessary to incorporate information about the relative positions of tokens within sequences so that the model can utilize sequence order effectively. To achieve this, "position encoding" is added to the Word2vec embedding output, forming the input for the encoder. The positional encoding method employed in this work was first introduced by Vaswani et al. [43] in a machine translation task.

The encoder is composed of a stack of N = 3 identical layers. Each layer has two sub-layers. The first sub-layer is a multi-scale self- and cross-attention network, while the second is a position-wise, fully connected feed-forward network. To facilitate effective information flow, each of these sub-layers incorporates a residual connection in conjunction with layer normalization.

The output generated by each sub-layer can be expressed as LayerNorm(x + sublayer(x)), where sublayer(x) represents the function associated with the sub-layer in question. Both the embedding layer and all model sub-layers yield outputs with a dimension of $d_{model} = 64$, allowing for seamless residual connections.

Upon completion of the classification process, a linear transformation followed by a sigmoid function is employed to convert the encoder output into predicted probabilities. We used grid-search to choose the hyperparameters on the training data of Chen et al., specifically, epoch in [50, 100], learning_rate in [5e−4, 5e−2], batch in [5, 10, 20, 60], and dropout in [0.2, 0.5]. Final epoch = 100, learning_rate = 5e−4, batch = 10, and dropout = 0.2 is the optimal hyperparameters.

### Multi-scale self- and cross-attention network

**Fig. 10:**
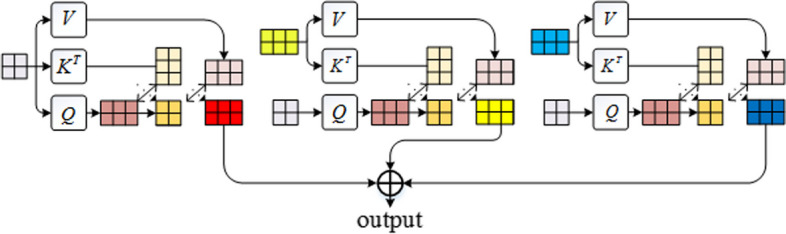
The internal structure of the multi-scale self- and cross-attention network

The multi-scale self and cross-attention network constitutes the initial layer of the encoder, designed to handle linguistic input at various scales. Utilizing word2vec embeddings, matrices at three distinct scales (take $X_{s0}^{{}} ,X_{s1}^{{}} ,X_{s2}^{{}}$ as an example) are introduced into the self-attention and cross-attention modules for simultaneous computation. Specifically, $X_{s0}$ is incorporated into the self-attention module, while the two combinations ($X_{s0}$ and $X_{s1}$, $X_{s0}$ and $X_{s2}$) are integrated into the cross-attention module. Subsequently, the outputs from these modules are directly added and relayed to the subsequent layer, as shown in Fig. 10.

### Cross-attention network

The cross-attention network is designed to extract and learn relationships between words in sequences of varying scales, effectively capturing associations across different sequences. Using sequences $X_{s0}^{{}}$ and $X_{s1}^{{}}$ as examples, we first transform each sequence into three different terms, which are query, key, and value. This is achieved through the application of linear projections.

7 $$Q_{s0} = X_{s0} W_{s0}^{Q} ,K_{s0} = X_{s0} W_{s0}^{K} ,V_{s0} = X_{s0} W_{s0}^{V}$$

8 $$Q_{s1} = X_{s1} W_{s1}^{Q} ,K_{s1} = X_{s1} W_{s1}^{K} ,V_{s1} = X_{s1} W_{s1}^{V}$$

where $X_{m} \in {\mathbb{R}}^{{m \times d_{model} }}$ is the output of the sequence embedding module, *m* represents the length of the input sequence $m \in \left\{ {s_{0} ,s_{1} ,s_{2} } \right\}$. $W_{m}^{Q} ,W_{m}^{K} \in {\mathbb{R}}^{{d_{model} \times d_{k} }}$, $W_{m}^{V} \in {\mathbb{R}}^{{d_{model} \times d_{v} }}$. *X*_*m*_ is transformed into the query matrix $Q_{m} \in {\mathbb{R}}^{{m \times d_{k} }}$, the key matrix $K_{m} \in {\mathbb{R}}^{{m \times d_{k} }}$, and the value matrix $V_{m} \in {\mathbb{R}}^{{m \times d_{v} }}$, in which *d*_*k*_ is the dimension of matrices *Q*_*m*_, *K*_*m*_, and *d*_*v*_ is the dimension of matrix *V*_*m*_.

Second, we compute the cross-modal dot product between the query vector of $X_{s0}^{{}}$ and the key vector of $X_{s1}^{{}}$, dividing the result value by $\sqrt {d_{k} }$, to estimate the association between the $X_{s0}^{{}}$ and $X_{s1}^{{}}$. These results are subsequently refined and normalized utilizing the softmax function, yielding attention weight coefficients. Lastly, we leverage these coefficients to aggregate the corresponding value vectors from each feature sequence, thereby facilitating that the associated information between the two sequences is obtained. The cross-attention function can be described as follows:

9 $$Cross - Attention(Q_{s0} ,K_{s1} ,V_{s1} ) = softmax\left( {\frac{{Q_{s0} K_{s1}^{T} }}{{\sqrt {d_{k} } }}} \right)V_{s1}$$

### Self-attention network

In contrast to the cross-attention module, which primarily focuses on inter-sequence interactions, the self-attention module identifies and elucidates intra-sequence associations. The self-attention function is described as

10 $$Self - Attention(Q_{s0} ,K_{s0} ,V_{s0} ) = softmax\left( {\frac{{Q_{s0} K_{s0}^{T} }}{{\sqrt {d_{k} } }}} \right)V_{s0}$$

### Multi-head multi-scale self- and cross-attention

The above elucidation pertains to single-headed attention, a fundamental mechanism in attention-based models. However, multi-headed attention is commonly employed in practice to augment model efficacy and expedite training. This technique entails conducting single-headed attention in parallel across multiple instances, known as "heads", and subsequently integrating the outcomes derived from each head. By incorporating multi-headed attention, the model can effectively capture diverse contextual information and intricate relationships inherent in the input data. The function of cross-attention is described as:

11 $$\begin{aligned} & MultiHead(Q,K,V) = Concat\left( {head_{1} ,...,head_{h} } \right)W^{O} \\ & \quad where^{{}} head_{i} = Attention\left( {Q_{s0} W_{s0s0i}^{{Q_{s0} }} ,K_{s0} W_{s0s0i}^{{K_{s0} }} ,V_{s0} W_{s0s0i}^{{V_{s0} }} } \right) \\ & \quad + Attention\left( {Q_{s0} W_{s0s1i}^{{Q_{s0} }} ,K_{s1} W_{s0s1i}^{{K_{s1} }} ,V_{s1} W_{s0s1i}^{{V_{s1} }} } \right) \\ & \quad + Attention\left( {Q_{s0} W_{s0s2i}^{{Q_{s0} }} ,K_{s2} W_{s0s2i}^{{K_{s2} }} ,V_{s2} W_{s0s2i}^{{V_{s2} }} } \right) \\ \end{aligned}$$

where the $W_{s0s0i}^{{Q_{s0} }} ,W_{s0s0i}^{{K_{s0} }} ,W_{s0s1i}^{{Q_{s0} }} ,W_{s0s1i}^{{K_{s1} }} ,W_{s0s2i}^{{Q_{s0} }} ,W_{s0s2i}^{{K_{s2} }} , \in {\mathbb{R}}^{{d_{model} \times d_{k} }}$, $W_{s0s0i}^{{V_{s0} }} ,W_{s0s1i}^{{V_{s1} }} ,W_{s0s2i}^{{V_{s2} }} \in {\mathbb{R}}^{{d_{model} \times d_{v} }}$ and $W^{O} \in {\mathbb{R}}^{{hd_{v} \times d_{model} }}$

In this task, we employ h = 8 parallel attention layers. For each layer,we use *d*_*k*_ = *d*_*v*_ = *d*_*model*_/h = 8.

### Position-wise feed-forward networks

After the multi-headed, multi-scale self- and cross-attention layer, a second sub-layer is incorporated to augment the representative capacity of the model further. This additional component comprises a position-wise, fully connected feed-forward network, enhancing the overall model performance. The architecture of this network entails two successive linear transformations, with an intervening rectified linear unit (ReLU) activation function, ensuring a non-linear and expressive representation of the input data. It is defined as:

12 $$FFN(x) = \max (0,xW_{1} + b_{1} )W_{2} + b_{2}$$

The input and output have dimensionality d_model_ = 64, while the inner layer's dimensionality is d_ff_ = 256.

### Classification module

To accomplish the classification task, the initial step involves computing the average of the encoder output. Subsequently, a linear transformation is applied, followed by implementing a sigmoid activation function. The optimization of the model is facilitated by employing cross-entropy loss as the primary objective. Finally, the methylation site probabilities are acquired, providing a robust and comprehensive representation of the underlying biological processes.

## Data Availability

The data supporting the findings of the article is available at the web server http://47.242.23.141/MSCAN/index.php.

## Abbreviations

RNA: Ribonucleic acid
m^1^A: N1-methyladenosine
m^6^A: N6-methyladenosine
Ψ: Pseudouridine
m^6^Am: N6,2′-*O*-dimethyl adenosine
Am: 2′-*O*-Methyladenosine
Cm: 2′-*O*-Methylcytidine
Gm: 2′-*O*-Methylguanosine
Um: 2′-*O*-Methyluridine
m^5^C: 5-Methylcytidine
m^7^G: 7-Methylguanosine
m^5^U: 5-Methyluridine
I: Inosine
CNN: Convolutional neural network
BiLSTM: Bidirectional long short-term memory
LSTM: Long short-term memory
RNN: Recurrent neural network
NLP: Natural language processing
DL: Deep learning
MSCAN: Multi-scale self- and cross-attention network
MCAN: Multi-scale cross-attention network
SCAN: Self- and cross-attention network
CAN: Cross-attention network
SAN: Self-attention network
Sn: Sensitivity
Sp: Specificity
ACC: Accuracy
Pre: Precision
MCC: Matthews correlation coefficient
AUROC: Area under the receiver operating characteristic
AUPRC: Area under the precision-recall curve
ENAC: Enhanced nucleic acid composition
